# Using a Human-Centered Design Approach to Determine Consumer Preferences for Long-Lasting Insecticidal Nets in Ghana

**DOI:** 10.9745/GHSP-D-18-00284

**Published:** 2019-06-24

**Authors:** Sharon Kim, Danielle Piccinini, Elorm Mensah, Matthew Lynch

**Affiliations:** a Johns Hopkins Carey Business School, Baltimore, MD, USA.; b Johns Hopkins Center for Communication Programs, Baltimore, MD, USA.; c URIKA Research, Accra, Ghana.

## Abstract

**Background:**

A human-centered design approach, paired with traditional research methods, was used to explore consumer preferences of middle-class Ghanaians for a long-lasting insecticidal net (LLIN) to be designed for the private-sector retail market.

**Methods:**

In March 2017, we conducted 9 focus groups with urban and rural middle-class Ghanaians across Ashanti, Greater Accra, and Western regions. A total of 78 participants (51 adults and 27 boarding school students) were involved in the focus groups. Participants were asked for their input on topics related to malaria prevention, LLIN perceptions and use behavior, as well as general consumer preferences related to the home and bedroom. They participated in a variety of exercises, such as rank ordering their preferences of various accessories that might be bundled with an LLIN and interacting with actual LLINs of different sizes and designs. The data were gathered and analyzed, using micro-interlocutor analysis framework to capture emergent themes.

**Results:**

LLINs are currently available through free distribution channels, but in most accounts, participants reported that the nets were inconvenient, uncomfortable, and not aesthetically pleasing, thus they were undesirable to use. For example, several participants described the process of hanging as well as entering and exiting the LLIN as challenging, stressful, and/or tedious. In addition, use of LLINs was considered to make people feel even hotter in an already warm climate as well as to leave users feeling confined within a small space. Finally, many participants discussed how to improve the look of LLINs including suggestions for additional colors, shapes, and hanging mechanisms to make the LLINs more compatible with their desired bedroom and home décor. Based on the participants' responses, we concluded that they would prefer LLINs that better reflect contemporary sensibilities. We devised and tested different LLIN attributes to address these points, focusing on a more convenient way to hang the net, a more attractive silhouette, and a zipper that allows the user to enter and exit with ease while still maintaining a sealed, mosquito-free space. A separate discrete choice experiment confirmed the attractiveness of these attributes by capturing the target population's willingness to pay for LLINs with said preference-congruent attributes.

**Conclusion:**

Our human-centered design approach yielded consumer insights and preferences for novel LLIN designs for the private-sector retail market in Ghana. If this net design is successful, it could increase LLIN use among the middle class and catalyze the purchase of LLINs to support ongoing malaria control efforts.

## Abstract (precis)

Through focus group discussions and human-centered design exercises, middle-class Ghanaians communicated the need to address convenience, comfort, and aesthetics when designing a bed net for their demographic. Illustrative attributes for consideration by private-sector manufacturers include a more convenient way to hang the net, a more attractive silhouette, and a zipper to provide ease of entry and exit while keeping the area sealed from mosquitos.

## BACKGROUND

The long-lasting insecticidal net (LLIN) is the mainstay of vector control for malaria prevention. Rapidly scaled-up dissemination through mass distribution of free LLINs has been credited with 68% of the 663 million clinical cases of malaria averted between 2000 and 2015.^1^ While the impact of widespread LLIN ownership and use has been substantial, a recent study of the LLIN use-access ratio, an indicator of usage given access to LLINs, has provided an additional important dimension to consider with regard to malaria prevention. Comparison of use-access ratios in Ghana show that among people who own LLINs, those in wealthier urban households have significantly lower LLIN use compared with those in rural households, particularly rural households in lower wealth quintiles.^2^

The reasons for inconsistent or non-use of LLINs vary and are not always fully known or understood. Understanding the barriers to LLIN ownership and use is an important factor in the allocation of resources for LLIN distribution by the National Malaria Control Program. If, for instance, the private sector can provide the middle class with affordable LLINs that meet consumer preferences, the National Malaria Control Program might be able to adjust its distribution efforts to allocate a greater number of free LLINs to populations at higher risk for malaria that use LLINs at higher rates.

In our work, we focus specifically on the LLIN usage of the middle class in urban and rural areas of Ghana. This demographic is growing and draws significant interest from public health advocates and researchers, among others, during the current period of unprecedented economic growth in Ghana.^3^ Our research is part of a larger project that has the overarching purpose of catalyzing a viable commercial market for LLINs. To help achieve this goal, we focused on the sector of the population that can afford to purchase an LLIN, the Ghanaian middle class. Understanding some of the reasons for lower use of LLINs despite access within this population might also help reveal promising ways to reduce the gap (e.g., via LLIN modification, LLIN diversification, distribution channel, LLIN promotions, social behavioral communication change efforts).

Understanding the reasons for lower use of LLINs despite access within a population might help reduce the gap.

In Ghana, mass campaigns typically distribute LLINs once every 3 years and are designed to achieve universal coverage by allocating 1 LLIN for every 2 people per household.^4^ During the periods between mass campaigns, LLINs are available through continuous distribution channels (e.g., certain services at health facilities) for biologically vulnerable populations, such as pregnant women when they attend their first antenatal visit and infants when they receive immunization for measles. LLINs are also provided through schools to students in grades P2 and P6.^5^ Should households not receive enough LLINs through these distribution channels, their options for obtaining an LLIN through other means are extremely limited. In other words, Ghana has no private-sector retail market for LLINs to speak of. According to a retail audit commissioned by the Private Sector Malaria Prevention Project,^6^ in 2017 only 7% of retailers in our 3 areas of focus (i.e., Ashanti, Greater Accra, and Western regions) sold bed nets. In addition, 95% of the total number of bed nets found on the market were claimed to be insecticide-treated, although the legitimacy of such statements was unverified and some of our interviewees questioned it. Many middle-class households in our areas of interest (approximately 82%) are using other malaria prevention methods such as aerosol insecticide sprays and coils.^6^

The LLINs that are available through public-sector distribution channels may vary in terms of shape, size, color, and material. In Ghana, these LLINs are commonly rectangular in shape and made to fit a double-size bed (approximately 190 cm × 180 cm × 150 cm). They are generally standard shades of white or blue in color and are made of insecticide-treated polyester or polyethylene.^7^ Notably, although there is some modest variety in terms of LLIN features, people do not get to choose what type of net they receive in mass campaigns or through continuous distribution channels. Upon receiving an LLIN, people are advised to hang it outdoors in the shade for at least 24 hours prior to using it. Once it has been aired out, the LLIN must be hung such that it covers the entire sleeping area. People usually tie strings to the loops in the top four corners of the net and connect them to nails or hooks in the ceiling, walls, or other supports.^8^ The standard rectangular LLIN is close-sided and must be fully tucked under the mattress or mat to create a sealed environment for effective vector control (Figure).^8^ Typically, users tuck the net most of the way around the mattress, leaving a space to crawl into bed, and then tuck in the open section from inside the net.

**FIGURE:**
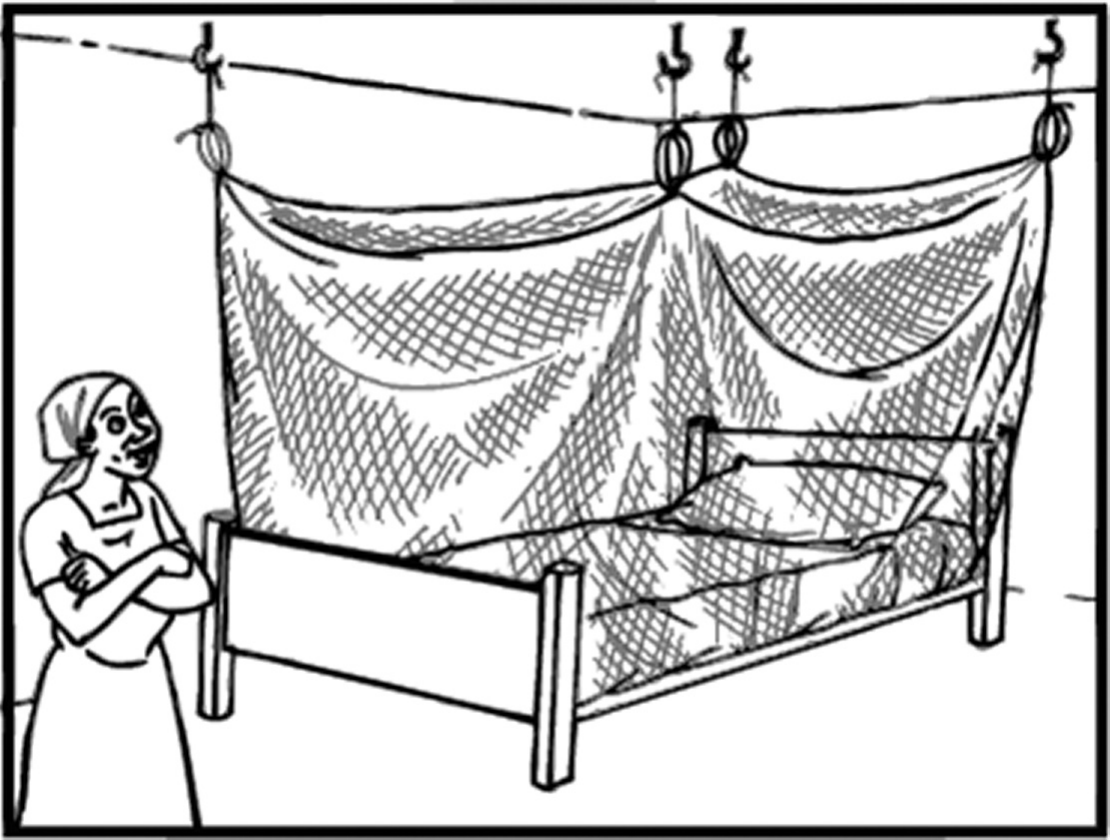
Illustration of a Rectangular Long-Lasting Insecticidal Net Properly Installed Over a Bed © 2009 International Federation of Red Cross and Red Crescent Societies.

Through a market analysis,^6^ we determined that LLINs designed for retail sale in Ghana would need to be noticeably different from the free LLINs distributed through public-sector channels, in ways that are attractive to target consumers. First, we found, unsurprisingly, that consumers are not likely to buy a product that is identical to one they can get for free elsewhere. Second, our target consumers were willing to pay for a differentiated LLIN provided it met their preferences. Our operating premise was that middle-class consumers will be more likely to use an LLIN that fits their stated consumer preferences and that regular use will protect users from malaria infection and reduce transmission rates by helping to control the mosquito population. Although attractive LLIN design options may be important for all users, our first goal was to help catalyze a retail market for LLINs among a population that can financially support it. Our work could be extended in the future to reach other users who were not represented in this single study.

Our operating premise was that people will be more likely to use an LLIN that fits their consumer preferences.

We wish to emphasize that our project pertained to LLINs only. Our research showed that a desire for malaria protection was a driver for purchasing prevention tools, and malaria prevention is very much a concern of middle-class Ghanaians. The responses indicated that many households consider the LLIN a desirable good but that it could be made more desirable. We feel a sufficient pool of demand exists in the middle class to drive the reestablishment of an LLIN market in Ghana. We expect that once this market is strengthened, it will expand to meet demand from other consumer segments of society over time. It should also be noted that the rationale behind these project efforts was not expected impact on disease burden among people in this segment of society (which would likely be small, given their lower risk), but rather building and expanding a sustainable market for LLINs that fully recovers costs.

Similar to other malaria prevention experts^9^ who have commented on the potential for redesign, we acknowledge the opportunity to put forth new LLIN designs to meet the contemporary needs of individuals living in malaria endemic areas. Thus, we sought to create new LLIN designs to meet one of our primary project goals of facilitating a functioning and competitive retail market for LLINs in Ghana. Our project focuses on the middle class because in order to catalyze a commercial market for LLINs, we first need to target the population with the economic means to purchase such a product. An in-depth exploration of the middle-class Ghanaian context was necessary in order to create LLIN designs that would meet that population's specific needs. To that end, we took a human-centered design approach to our market research and product development efforts.

## METHODS

### Setting

The bulk of this work was conducted in Ghana in the Ashanti, Greater Accra, and Western regions, with some work conducted in Baltimore, Maryland (USA). These areas of Ghana are ideal locations to sell LLINs because malaria is endemic and there are proportionately lower levels of poverty. In other words, a significant number of people in these areas are at risk of malaria and can afford to buy commercial LLINs.

### Human-Centered Design Process

Human-centered design (HCD) is an approach to creative problem solving that prioritizes direct engagement with various stakeholders to glean insights that may be critical to designing products that are both novel and useful to a given market or audience; HCD also endorses iterative prototyping and testing.^10^ HCD is considered an umbrella category of various design approaches (e.g., user experience design, design thinking) and is a well-established part of certain business practices, such as product design and development. More recently it has begun to take hold in other professional sectors, including public health (e.g., see the Design for Health Initiative by the U.S. Agency for International Development and the Bill & Melinda Gates Foundation).^11^ Although inherently dynamic and iterative, the HCD process is often described in a series of simple steps (Table 1).^12^ We provide these steps as a reference, highlighting that it is a common way to frame this type of work; however, it is important to note that the steps are not executed as cleanly in reality as the table might suggest. For instance, the *Empathize* work, when the team immerses itself in the context of the key stakeholders, not only happens at the beginning but ideally is present throughout the other steps as both a guiding principle and a “check and balance” of human-centeredness.

**TABLE 1.** Steps of Human-Centered Design Process

Empathize	Define	Ideate	Prototype	Test
Conduct secondary research Identify key stakeholders Engage with them in various ways to understand their circumstances and psychology	Identify insights to refocus and redefine the challenge at hand	Generate ideas and solutions	Create low-fidelity prototypes of ideas for testing	Test prototypes to gain useful information Use that information to continue refining ideas and solutions

Human-centered design prioritizes direct engagement with various stakeholders.

Other user-centered approaches such as Trials of Improved Practices have been featured in selected public health projects, for instance, in efforts to improve nutrition planning^13^; however, we specifically chose to apply HCD because it allowed us the greatest flexibility in our transdisciplinary approach to working with our key stakeholders. Further, it is a practice that was familiar to our partners in both the private and public sectors. We used HCD in conjunction with traditional methods of quantitative and qualitative research (e.g., household surveys and focus group discussions [FGDs]). The purpose of this mixed-methods approach was not only to capture existing consumer preferences but also to tap into or discern latent preferences for a consumer product that does not yet exist. In other words, we needed to have a solid understanding of the consumer psychology within this population in order to create new LLIN designs that would be desirable to it. For this purpose, HCD was especially helpful because it encouraged individual participants to express thoughts and feelings about their experiences related to LLINs and malaria that would be more difficult to capture in other ways. For example, exercises and activities were designed to allow participants to brainstorm, imagine, and share intimate details regarding their domestic behaviors and routines. The result was a rich mix of data and the identification of key consumer insights regarding middle-class Ghanaians' perceptions of self, their behaviors and attitudes related to malaria prevention, and their use of LLINs.

A solid understanding of the consumer psychology within the study population was needed to design a desirable LLIN.

#### Literature Review

The *Empathize* phase of our HCD work began with an orientation to our target population and their current malaria prevention practices. We conducted secondary research including a targeted scan of peer-reviewed and gray literature related to malaria prevention, LLINs, and associated development and public health initiatives in Ghana. To further contextualize this research, we conducted the aforementioned market analysis as well as a series of supplementary interviews with key stakeholders. Our explicit goal was to gain a deeper understanding of the contemporary attitudes regarding these topics as well as the human experiences related to them, which could not be gleaned directly from the literature review.

#### Market Analysis

The market analysis included both qualitative (key informant interviews with different members of the supply chain) and quantitative (household surveys, discrete choice experiments, and retail audits) work.^6^ Through a combination of key informant interviews with 12 members of the LLIN supply chain and 271 retail audits, we sought to gain a better understanding of any reluctance to invest in an LLIN retail market in Ghana. The most commonly mentioned reason for a lack of interest was weak consumer demand. Owing to this low demand, manufacturers tended to focus their sales efforts on large institutional buyers like the Ministry of Health and NGOs, whereas distributors and retailers put their efforts toward importing, stocking, and selling fast-moving consumer goods, which do include some mosquito control products (e.g., insecticide sprays and coils). Manufacturers and distributors were also specifically concerned about competition from counterfeit LLINs and LLINs leaked from free distribution channels. Despite these concerns, LLIN supply chain personnel were generally excited about the opportunity to catalyze a new market.

We sought to gain a better understanding of any reluctance to invest in an LLIN retail market in Ghana.

#### User Interviews

For our supplementary interviews with key stakeholders, we asked our subjects, including local community experts, consumer product managers, and malaria project team members (based in Ghana and the United States), to describe their own experiences with LLINs and why they did or did not use them. For example, one person described not using an LLIN because she had easy access to antimalarial drugs. Another described how the fear of scorpions and other vermin motivated him to use an LLIN regularly to get a good night's rest. Although this information was not necessarily generalizable to a wider population, the interviews helped us unpack the psychology of an LLIN user of some financial means. In capturing and reviewing their responses, we began to consider what other social determinants of LLIN use we should investigate related to our project.

#### Stakeholder Analysis

With this snapshot of the environment and general circumstances in mind, the project team, which consisted of the facilitators of the HCD work, supporting malaria team members, and selected individuals who represented the demographics of the target market (e.g., middle-class Ghanaians), completed an extensive stakeholder analysis. A visual documentation of all stakeholders related to the project (primary [e.g., local retailers], secondary [e.g., health organizations that distribute free LLINs], and peripheral [e.g., local and international influencers]) as well as their relationships to each other was created in the form of a stakeholder map. Through this exercise, we were able to visualize potential partners, opportunities, and barriers in the existing system. We then conducted a series of live and video conference interviews, brainstorming and prototyping sessions with middle-class Ghanaians who had varying levels of experience with malaria and bed nets. These sessions yielded some initial ideas that we planned to test in-country with FGDs attended by individuals from our target population.

Through a stakeholder analysis, we visualized potential partners and opportunities as well as existing barriers.

#### Focus Group Discussions

With this information in mind, we planned our FGDs, modified with HCD exercises, to be held in Ghana. Nine FGDs were conducted in March 2017, in the Ashanti, Greater Accra, and Western regions of Ghana. Our vision was to facilitate a series of activities designed to capture the thoughts, feelings, and opinions of urban and rural middle-class Ghanaians related to malaria prevention, LLINs, and their consumer preferences (e.g., preferred colors for the home and bedroom). People participated in a variety of exercises designed for this purpose. For example, in one exercise, participants were asked to rank order their preferences of various accessories that might be bundled with an LLIN (e.g., adhesive hooks), using a stack of cards with images of the accessories on them. In another experiential exercise, stations were created to observe people interacting with actual LLINs of different sizes and designs that had been hung around the venue. We noted common behaviors such as cautious sniffing of the LLINs and continuous patting and “fluffing” of the LLIN from the inside. The information gathered from these activities coupled with data collected from our FGDs helped us better understand how we might improve upon existing LLINs by creating designs that would be appropriate, as well as desirable, for this group of people.

Although the FGDs were designed and facilitated as a part of a cohesive suite of HCD activities executed throughout the overarching project, we have chosen to highlight these specifically because they yielded some important insights that subsequently influenced the features of our preliminary LLIN design prototypes. HCD is inherently transdisciplinary, and in our research and practice, we have found that its execution can be highly dependent on the implementer. From our perspective, the benefit of using a method like FGDs in an HCD context is that it can be used more flexibly than approaches that are wedded to a single disciplinary approach or formal research guidelines that place an overarching priority on standardization and protocol. For us, this meant allowing facilitators and interviewers the freedom to explore and follow unexpected or interesting threads in the conversations. It also allowed us to incorporate less conventional activities (e.g., expressing oneself through creative activities).

Importantly, the FGDs yielded insights that influenced the design of our LLIN prototypes.

### Recruitment of Participants

We used a nonprobability sampling approach, or convenience sampling, to recruit participants for the FGDs. The total sample included 78 participants (51 adults and 27 students).

The study included 2 main target groups, namely:

- Middle-class adults (ages 18–59) who possessed the disposable income necessary to afford purchasing LLINs from the retail market.
- Senior high school students (ages 15–17) in boarding schools who were required to bring LLINs to their boarding houses, as indicated on their prospectus.

A standard screener, in the form of a questionnaire, was administered to identify participants who met the qualifications for participation, which involved previous experience using bed nets, income level (lower-middle to upper-middle class), profession, employment status (e.g., employed, not employed, boarding school student), and age. This screener helped us achieve adequate representation of our groups of interest. Participant demographics are presented in Table 2.

**TABLE 2.** Demographic Summary of Focus Group Discussion Participants, by Group

Group	Total No. of Participants	Region of Ghana	Area	Population Segment	Gender Composition (%)	
					Male	Female
1	8	Western	Rural	Adult (ages 18–58)	62.5	37.5
2	10	Western	Rural	Student (ages 15–17)	60.0	40.0
3	8	Western	Urban	Adult (ages 18–58)	50.0	50.0
4	8	Greater Accra	Urban	Adult (ages 18–58)	50.0	50.0
5	8	Greater Accra	Rural	Adult (ages 18–58)	50.0	50.0
6	9	Greater Accra	Urban	Student (ages 15–17)	55.6	44.4
7	9	Ashanti	Urban	Adult (ages 18–58)	66.7	33.3
8	8	Ashanti	Urban	Student (ages 15–17)	37.5	62.5
9	10	Ashanti	Rural	Adult (ages 18–58)	50.0	50.0

### Procedures

Our FGDs employed a conversational style of group interviewing to explore issues related to experience with malaria, perceptions of malaria, methods for malaria prevention, and facilitators and inhibitors of LLIN use. Each FGD had 2 facilitators, one of whom was proficient in the local language, and 2 notetakers. Facilitators were provided with loose scripts to ensure that certain topics were covered in all sessions. FGDs were conducted in English unless it was decided that the local language was more appropriate.

FGDs enabled exploring experience and perceptions of malaria, methods for malaria prevention, and facilitators and inhibitors of LLIN use.

Our FGDs included the following exercises. Participants were observed and their reactions and comments were captured via audio recorder and by a notetaker during discussions and on video and by a notetaker during experiential activities (with participant permission per Institutional Review Board requirements):

- **Discussions:** Participants were asked to share their thoughts, feelings about malaria, net use, and current net designs. They were also asked for their suggestions on what design aspects would improve their chances of buying and using an LLIN.
- **Color/design preference activity:** Participants indicated their preferred color and design preferences for a bed net. They were provided with a black and white drawing of a bed net (various shapes) and asked to use art supplies to indicate any preferred color choices or designs (e.g., logos, patterns).
- **Net material preference activity:** Participants were given swatches of 3 different materials (polyester, polyethylene, or polypropylene) currently being used for LLINs and asked to describe their reactions to touching them and discuss their preferences and perceptions of the materials.
- **Bundling preferences:** Participants were shown cards with a selection of hypothetical accessories that could be bundled with a bed net to gauge their desire and preferences for such products. They included items such as adhesive hooks and small fans.^14^ These items were chosen because they emerged in our research and initial interviews as potentially attractive products to bundle with an LLIN that might encourage purchase. Participants were asked to sort the cards in order of preference and discuss their reasoning aloud. Card sorting was used because it can help participants prioritize preferences more easily than using pen and paper or a drop-down menu on an online survey.
- **LLIN experiential activity:** Nets of various shapes and sizes were set up around the workspace. Participants were observed while they examined, entered, spent some time inside, and exited the nets. They were invited to describe any of their feelings about the experience to facilitators and notetakers.
- **Time to reflect and conclusions:** Participants were given an opportunity to ask any remaining questions or share any final thoughts before departing.

### Data Analysis

Data captured from the FGDs were transcribed and analyzed together with additional field notes taken by the facilitators and notetakers. We used the micro-interlocutor analysis framework^15^ for qualitative analysis of these data to capture emergent themes. Compared with other methods of FGD data analysis, this framework allowed us to consider the voices, or lack thereof, of all participants in the insight generation process by analyzing the answers given and not given by each participant instead of seeking or focusing primarily on points of group consensus.^16^

## RESULTS

Three main constructs—convenience, comfort, and aesthetic—emerged from our analysis regarding key barriers to LLIN use as described by our participants (Table 3). The barriers were related to use of a double-size rectangular LLIN with 4 hanging points, which is the most common type of LLIN distributed in Ghana through the public sector. With regard to convenience, several participants described the process of hanging as well as entering and exiting the LLIN as challenging, stressful, and/or tedious. Another issue was the lack of access to things around the bedroom, such as eyeglasses, phones, bibles, and condoms, while using the LLIN. Participants were also concerned about discomfort associated with using the LLIN. Specifically, use of LLINs was considered to make people feel even hotter in an already warm climate as well as to leave users feeling confined within a small space. Multiple participants mentioned that using an LLIN was not conducive to having sex for similar reasons. People also complained about the polyethylene material of the LLIN saying it was “hard” and rough to the touch, and some expressed concerns regarding the insecticide treatment and perceived side effects associated with its use (e.g., burning sensations in the eyes or on the skin). Finally, participants also discussed how LLINs were aesthetically incompatible with their desired bedroom and home décor. Many discussed their thoughts on how to improve the look of LLINs including suggestions for additional colors, shapes, and hanging mechanisms, among other design modifications.

**TABLE 3.** Key Barriers to LLIN Use According to Focus Group Discussions

Construct	Barriers to Use	Selected Quotations	Examples of Suggested Improvements
Convenience	Hanging: Tedious to affix or hang because of multiple (at least 4) hanging points Challenges with finding accessories needed for hanging (e.g., nails, hooks, poles, and strings) Concerns regarding defacement of bedroom walls by drilling nails or hooks that serve as hanging points	“Bed net installation is stressful.” —Male Adult, Rural, Ashanti Region	LLIN with minimal hanging points (e.g., single point hang) Provision of hooks (regular or adhesive) and string with LLIN
	Entry and exit: Not easy to enter and exit the LLIN—users have to lift them over their heads each time they enter or exit as well as tuck them under the mattress once inside	“In the middle of the night, when I have to pee, I have to think hard about it, should I wait until morning, or should I go through the stress of getting out of my net?” —Female Student, Urban, Greater Accra Region	A mechanism that does not require the LLIN to be untucked and lifted overhead for entry and exit (e.g., overlapping flaps or zippered entry in the side of the LLIN)
	Access to personal items: When inside the LLIN, users are restricted from accessing nearby personal items that are outside the net (e.g., things on a bedside table, such as bible, phone, eyeglasses, water, condoms)	“As a mother with a baby, I can keep baby pampers in the pocket.” —Female Adult, Rural, Greater Accra Region “With a pocket in the bed net, I can put my bible and notebook in there.” -Male Adult, Rural, Western Region	A pocket inside the LLIN for storage of items that may be needed during the night Improved entry and exit also addresses this barrier
Comfort	Restrictive space: The feeling of being closed-in (e.g., claustrophobic sensation) or lack of space within the LLIN Particularly an issue for sexually active couples	“I am married and I can't see myself playing games with my wife in a bed net. Even when I was single the bed net was not spacious for me alone. Imagine now that I am married, it won't work unless it is made spacious, so we can feel free in it.” —Male Adult, Urban, Ashanti Region	Rectangular LLINs as they are perceived as more spacious LLINs in various bed sizes
	Heat: Weather conditions are too hot to use an LLIN comfortably Perception that LLINs increase the heat felt while sleeping	“The weather, the weather, the weather. It is just hot for bed nets! Especially when you use those hard [polyethylene] nets.” —Male Adult, Rural, Western Region	Provide a small fan to reduce the heat (e.g., Briët et al.^14^)
	Material texture: The material texture of polyethylene LLINs are perceived as both rough and hot	“The [polyethylene net] is hard and can give skin rashes.” —Male Student in Urban, Ashanti Region “I didn't like the [polyethylene net] because it feels like rubber, so if you use it, there would be a lot of heat.” —Female Adult, Urban, Greater Accra	Use polyester material for LLINs as it is perceived to be softer and less hot
	Insecticide treatment: Adverse reactions to the insecticide in LLIN (e.g., skin irritation and itchiness)	“The real problem is the chemicals in the net. If we use it and it makes us uncomfortable then we stop using it.” —Female Adult, Urban, Greater Accra	Improved messaging about proper care of LLINs before first use (e.g., air out LLIN for at least 24 hours before using)
Aesthetics	LLINs detract from bedroom décor by creating a cluttered look in the bedroom Limited options of LLIN styles and sizes that complement bedroom décor.	“The nets make your room look ugly. We are forced to use it because it protects us against mosquito bites. We need fancy nets, with a variety of colors we can choose from, and ones that are nice to hang so we can do away with all the poles and nails in the walls.” —Female Adult, Rural, Greater Accra	An LLIN design that is more sleek (e.g., minimal hanging points) LLINs available in a variety of sizes and colors

Abbreviation: LLIN, long-lasting insecticidal net.

Our analysis revealed that key barriers to LLIN use pertain to convenience, comfort, and aesthetics.

The participants provided us with a great deal of information about their lifestyles and LLIN preferences. More importantly, their responses support our view that an opportunity exists to modify the standard LLIN design to satisfy the contemporary consumer preferences of the Ghanaian middle class. We used these emergent constructs as the basis for developing differentiated LLIN designs that would be more desirable to this target population.

### Key Insights

We found that LLIN usage gaps among our middle-class Ghanaian participants may exist to some degree because of evolving consumer preferences. From what we gathered, their current preferences reflect a desire for things that match contemporary sensibilities (e.g., convenience, comfort, and personal style) and reflect an upwardly mobile lifestyle. We found that trade-offs are made on functional effectiveness and affordability for a mosquito control product that is easier to use. This finding may explain why an overwhelming majority of our target population preferred non-LLIN malaria control products (e.g., aerosol insecticide sprays and coils) despite acknowledging that LLINs are more effective for malaria prevention and less expensive. As previously mentioned, these alternative forms of mosquito control are quite prevalent among this demographic and compete directly with LLINs because they are affordable, convenient, and, we posit, perceived to be a more modern approach to malaria prevention than LLINs, which carry a different connotation. We argue that in order for LLIN use to increase among this population, the design of LLINs needs to change. The new design(s) must be convenient, comfortable, and aesthetically pleasing, while also maintaining the key functionality of protecting against mosquito bites.

Changing or tweaking certain LLIN features could aid in demedicalizing their use, thus positioning LLINs to become something more desirable to use in the home. To date, LLINs have been positioned as a tool almost entirely for malaria prevention purposes and have been distributed with a 100% subsidy (free of charge) like many other health commodities. Our goal in introducing the concept of differentiated LLINs was to create a product that is convenient for use, similar to the non-LLIN mosquito control products, and also to position it as something that would not detract from, and might even improve, one's interior design. Additionally, LLINs currently on the market are primarily sold through pharmacies with little demand, and our research suggests that there might be a repositioning opportunity by selling LLINs in other, nonmedical, retail outlets (e.g., supermarkets, convenience shops).

### Prototype Development and Testing

With an understanding of these 3 barriers to LLIN use, the study team, along with members of the target market (a panel of middle-class Ghanaians), devised and tested different LLIN attributes that address these drivers. In keeping with HCD best practices, the insights yielded from our in-country work were thoroughly reviewed by our project team and became fodder for several brainstorming sessions on how to best meet the stated consumer preferences of our target populations within our constraints (e.g., maintaining vector control efficacy, economic factors). Our intention was to use our learning to create prototypes that addressed the barriers and pain points indicated by our participants. After much discussion, we prioritized what we felt were the most important (and feasible) LLIN design features, which ultimately included a more convenient way to hang the net, a more attractive silhouette, and a zipper that allows the user to enter and exit with ease while still maintaining a sealed, mosquito-free space. A separate discrete choice experiment confirmed the attractiveness of these attributes by capturing the target population's willingness to pay for LLINs with said preference-congruent attributes.^17^

Based on the study findings, we concluded the most important bed net features included a more convenient way to hang the net, a more attractive silhouette, and a zipper for ease of entry and exit.

We have since shared our consumer insights and preliminary ideas for new design features with current manufacturers globally who supply LLINs in Ghana. We hope these partners take this information into consideration as they make decisions about current and future LLIN supply, demand, and marketing and will pursue pilot testing of new net designs for the private-sector retail market in Ghana. Should that opportunity come to fruition, we intend to support efforts to test the ideas in-country to continue improving designs for the maximal benefit of our target population. This iterative approach to solution refinement directly reflects the tenets of HCD.

## DISCUSSION

Human centeredness should not be thought of as a binary designation but rather a feature or quality upon which to pursue continuous improvement. In other words, achieving human centeredness in public health services and interventions does not have to have a ceiling. Moreover, just because HCD is suggested as an approach does not necessarily mean that the target of these efforts was not originally created with key stakeholders or users in mind. For example, in the case of this project, it was suggested that HCD could take a medically effective product and make it more desirable to use among a population that had experienced an immense amount of societal and economic change since the product was first designed. An HCD approach was pursued because it was appropriate and ideal for the type of information we needed to achieve this goal.

One of the most prominent lessons we learned from this work is the importance of contextualizing the use of public health interventions so they are tailored to specific settings and populations. It is unlikely that any single LLIN design can serve all people equally well. Our work confirmed what we already knew—even within a single country like Ghana, people are not monolithic. To serve the needs of the middle class in the urban and rural areas in the course of catalyzing a commercial market for LLINs, interventions should be considered in the overall context. Specifically, the LLIN can be thought of not only in terms of vector control and malaria prevention but also as a consumer product that may be used or not used because of how it makes people feel about themselves and their homes. This approach is important for public health professionals because the LLIN is currently the most effective form of malaria control, but it has competition from other, less medically effective products that are viewed as consumer/home goods.

One of the most prominent lessons learned is the importance of contextualizing the use of public health interventions.

We also discovered that prevention of malaria competes with other values and objectives, such as convenience, comfort, and aesthetics. When researchers consider LLINs, they may not necessarily view them in relationship to use of bibles and condoms kept on nightstands; however, our target population does. People care about their health, but they also care about sex and romance, their religious practice, the look and feel of their homes, and personal comfort, among other things. We saw these priorities as potentially being in competition, so it is important to consider how to accommodate them in new LLIN designs. We believe these kinds of discoveries will more readily emerge from taking an HCD approach because a more traditional research approach may require a narrower focus or greater adherence to standardization and clear-cut methods. HCD helped us to identify the needs and wants of a population that has experienced a great deal of change in the last couple of decades. It also helped us to glean consumer preferences for a product about which there is almost minimal knowledge of consumer preferences and to design a medical product that may also be commercially desirable. Human centeredness was at the core of each of these questions. The flexible, personal, user-centered approaches of HCD made sense for us and provided advantages over other traditional research methods.

People care about their health, but they also care about sex, romance, religion, their homes, comfort, and other things.

The introduction and execution of self-expressive and experiential exercises within the context of FGDs significantly improved the richness of our data. The inclusion of these activities helped set a tone for the day of work together that promoted candid sharing and creativity. Additionally, the underlying philosophy of HCD as an experimental, iterative form of problem solving encouraged the project team and the stakeholders to play with their ideas and collaborate across groups and teams. Gleaning the same insights or soliciting the same level of engagement through traditional approaches is difficult to imagine.

We were surprised at how pleased our participants were to be consulted on the design of a product for them. One female participant in the rural area of Ashanti Region remarked:

*We always have free bed nets given to us, or have to buy [sic] whatever bed nets shops have available to sell, yet no one has ever taken the time to come ask us what we want in a bed net. So thank you for asking us that today and taking our opinions into consideration!*

For us, this comment was noteworthy. We initially decided upon an HCD approach to ensure fidelity to our target population's needs and interests. We did not necessarily expect that engagement in the process would also be meaningful to participants.

For the portion of our work that we chose to highlight in this article, our project team selected an approach that aligns closely with qualitative research methods. It made sense for us given our project goals and our team's training and experience. It is important to note that HCD does not promote any single academic perspective, methodology, or approach over others. HCD is practiced differently depending on the composition and collective expertise of the team and the problem or challenge that it seeks to address. We believe this openness and flexibility can be a tremendous advantage for those who seek permission to explore innovative and/or cross-collaborative methods and approaches to their work. We believe our team's experience can be learned from, but we do not submit our work as a template for all global health HCD projects.

The increasing number of public health projects that use HCD^18^ appears to connect more broadly to a larger trend of human-centered initiatives in health care. The concept of health and well-being today is more nuanced such that hospitals, care providers, medical technology companies, and others are realizing that they can no longer afford to focus solely on the primary health goal. They must also consider how social determinants of health influence behaviors and intervention uptake. For this project, we realized we had to expand our thinking beyond the preventative medical aspects of the LLIN in order to meet the research objective of catalyzing a commercial market for LLINs and ultimately the goal of increasing malaria prevention among our target population. For a health technology such as the LLIN to produce a benefit, it has to be used. Therefore, public health professionals should pay serious attention to the factors that drive or constrain use, including the perceptions and preferences of end-users.

## Notes

**Peer Reviewed**
